# How Does Epstein–Barr Virus Contribute to Chronic Periodontitis?

**DOI:** 10.3390/ijms21061940

**Published:** 2020-03-12

**Authors:** Kenichi Imai, Yorimasa Ogata

**Affiliations:** 1 Department of Microbiology, Nihon University School of Dentistry, Chiyoda-ku, Tokyo 101-8310, Japan; 2 Department of Periodontology, Nihon University School of Dentistry at Matsudo, Chiba -271-8587, Japan; ogata.yorimasa@nihon-u.ac.jp

**Keywords:** Epstein–Barr virus, chronic periodontitis, periodontopathic bacteria, microbial interaction, periodontopathic virus

## Abstract

Chronic periodontitis is spreading worldwide and mutually interacts with systemic diseases like diabetes mellitus. Although periodontopathic bacteria are inevitable pathogens in their onset and progression, many cases are not ascribable to the virulence of these bacteria because the effect of plaque control is limited. In contrast, Epstein–Barr virus (EBV) in the periodontium has been correlated with chronic periodontitis and has recently been considered as a promising pathogenic candidate for this disease. However, several important questions have yet to be addressed. For instance, although EBV latently infects more than 90% of individuals over the world, why do patients with chronic periodontitis exclusively harbor progeny EBV in the oral cavity? In addition, how does latently infected or reactivated EBV in the periodontium relate to the onset or progression of chronic periodontitis? Finally, is periodontitis incurable because EBV is the pathogen for chronic periodontitis? In this review, we attempt to answer these questions by reporting the current understanding of molecular relations and mechanisms between periodontopathic bacteria and EBV reactivation in the context of how this relationship may pertain to the etiology of chronic periodontitis.

## 1. Introduction

Epstein–Barr virus (EBV), a gamma-herpesvirus, latently infects more than 90% of adult humans worldwide [1]. However, some cases of EBV infection cause clinical manifestations including infectious mononucleosis, autoimmune disorders, and a number of malignancies including Burkitt’s lymphoma, Hodgkin’s disease, nasopharyngeal carcinoma, and gastric adenocarcinoma [1]. Like other herpesviruses, EBV establishes a persistent infection in the host during which it has latent and lytic phases [1,2]. EBV is transmitted from person to person via saliva and passes through the oropharyngeal epithelium to B lymphocytes, where it establishes a lifelong latent infection but is sometimes reactivated unpredictably to cause life-threatening diseases [1,2]. Elucidation of the molecular mechanisms involved in maintaining and disrupting EBV latency (EBV reactivation) has therefore been a central topic of EBV research.

**Figure 1:**
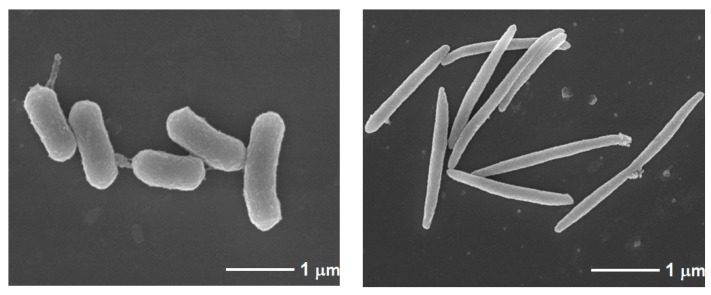
Electron microscopic image of *Porphyromonas gingivalis* and *Fusobacterium nucleatum.* (**A**) *P. gingivalis* is an oral Gram-negative black-pigmented strict anaerobic bacterium. It is frequently found in the plaque biofilms on tooth surfaces from individuals with periodontal diseases. *P. gingivalis* is implicated in periodontal disease and systemic diseases. Its pathological effects are mediated by a variety of virulence factors including lipopolysaccharide (LPS), fimbriae, proteases, and short-chain fatty acids such as butyric acid (BA). (**B**) *F. nucleatum* is a Gram-negative anaerobic rod bacterium and a common resident of the human mouth and gut. This bacterium causes a wider variety of inflammatory diseases such as periodontitis, appendicitis, inflammatory bowel diseases, and colorectal cancer.

Chronic periodontitis, a complex, chronic inflammatory disorder that involves interactions of specific bacterial pathogens and host cellular responses, is among the most prevalent microbial diseases in humans [3,4]. Severe chronic periodontitis results in the loosening of teeth, occasional pain and discomfort, impaired mastication, and eventual tooth loss. Specific bacterial species, mostly Gram-negative bacteria such as *Porphyromonas gingivalis*, *Fusobacterium nucleatum*, *Tannerella forsythia*, and *Treponema denticola* show a close association with periodontitis (Figure 1) [3,4].

Infection with periodontopathic bacteria and their released surface cellular structures such as lipopolysaccharides (LPSs) and fimbriae stimulate host immune responses and result in the production of inflammatory mediators and matrix metalloproteinases, which leads to connective tissue destruction and bone resorption in the periodontium [3,4]. In addition, over the past two decades, chronic periodontitis has been recognized as a risk factor for several systemic pathologies such as heart disease, diabetes mellitus, and pre-term birth [3,4]. In this context, we have previously reported that chronic periodontitis may contribute to AIDS progression [5,6], where human immunodeficiency virus (HIV) is involved as a latently infected agent in the host cells and is reactivated with the metabolic activity of co-infected periodontopathic bacteria. These findings have implied that periodontopathic bacterial infections may be direct or indirect causative factors in numerous systemic diseases.

On the other hand, although it is generally believed that chronic periodontitis is caused by periodontopathic bacteria, the onset and progression of a few cases are difficult to explain as ascribable to the bacteria alone. Specific pathogens sometimes cannot be detected in the periodontal flora, and the composition of periodontal flora does not always differ from that of normal healthy flora [7,8,9]. Furthermore, periodontal treatment often does not help. Therefore, it is hypothesized that bacteria are necessary only for the initiation of chronic periodontitis as a trigger. In our recent understanding of chronic periodontitis etiology, the induced disorder or deterioration of host immunity has more importance for manifestations of this disease. In this regard, the ability of EBV to suppress host immunity has attracted much attention, including ours, to the consideration of EBV as a causative agent for periodontitis. Representative studies indicate this causative relationship between chronic periodontitis and EBV. First, more EBV DNA is found in gingival crevicular fluid and saliva of periodontal patients than in otherwise healthy control groups [10,11,12,13,14,15,16]. Second, EBV prevalence in patients with chronic periodontitis has correlated with periodontal pocket depth [15,16,17]. Third, bacterial and viral co-infections are more frequent in deep periodontal pockets, where *P. gingivalis*, *T. forsythia*, EBV-1, human cytomegalovirus, *Aggregatibacter actinomycetemcomitans*, and EBV-2 are detected in 95%, 75%, 72.5%, 50%, 12.5%, and 10% of sites, respectively, with probing pocket depths deeper than 6 mm [18]. Fourth, higher densities of *P. gingivalis* are also found in EBV-positive periodontal patients [13,18,19]. Fifth, antiviral treatment has resulted in decreased EBV detection and an improved periodontal condition [20]. Sixth, EBV-positive B lymphocytes and EBV-positive gingival epithelial cells are verified in the periodontium with chronic periodontitis [15,17]. These observations suggest a causative relationship between chronic periodontitis and EBV as well as periodontopathic bacteria. Therefore, a new term “periodontopathic virus” has finally emerged.

Although this evidence has implicated EBV in the onset and/or progression of chronic periodontitis, the questions of why much more EBV is detectable in patients with chronic periodontitis than periodontally healthy subjects, how latent EBV is reactivated in the periodontium, and how released EBV contributes to the onset and progression of chronic periodontitis remain unclear.

In the present review, we report the current understanding of molecular mechanisms of how periodontopathic bacteria reactivate latently infected EBV in the periodontium. In addition, we discuss how this may pertain to the etiology of chronic periodontitis. Finally, taking EBV infection into account, we propose an attitude that all health care workers including dentists should take when they face chronic periodontitis.

## 2. Molecular Mechanism of the Maintenance of EBV Latency

Epigenetic regulation such as post-translational modification of DNA-associated histones ubiquitously occurs in cells while genes function [21,22]. During this regulation, several chemical modifications are involved such as acetylation, methylation, phosphorylation, and ubiquitination [21,22]. These modifications play an important role to control the conformation of chromatins and their transcriptional status [21,22]. Of clinical importance, this regulation is involved in the pathogenesis of a broad range of diseases such as cancer and microbial infections [21,22]. Particularly, lysine acetylation by histone acetyltransferases (HAT) including cyclic AMP-responsive element binding protein (CBP) and p300, and deacetylation by histone deacetylases (HDACs) play central roles in viral latency and reactivation [21,22].

For example, in the case of HIV, HIV-1 gene expression is regulated by histone modification during the lytic and latent stages of infection [23]. We and others have reported that transcriptional repressors recruit HDACs to the 5′ long terminal repeat of HIV-1 and consequently maintain HIV-1 latency by repressing transcription of HIV-1 proviruses [23,24]. By contrast, activation of HIV-1 gene expression by cell stimulation induced by mediators such as HDAC inhibitors and tumor necrosis factor-α (TNF-α) is correlated with local histone acetylation, which dismisses the negative regulator/HDAC protein complexes, thus initiating transcription [5,6,23]. In addition, a switch between the lytic and latent stages of herpesvirus infection is also determined by the viral chromatin status [2,25]. Furthermore, hepatitis B virus replication is also associated with specific epigenetic marks, such as histone acetylation or deacetylation [26].

In the case of EBV, it is usually stable during lifelong latency in host cells. However, when the transition occurs from the latent to the lytic replication cycle in latently infected cells, it is regulated by an epigenetic “on” and “off” modulation representing “open” and “closed” conformations of chromatin, as is observed in other viruses mentioned above [2,27]. The product of the BamHI Z fragment leftward open reading frame 1 (BZLF1) gene (BamHI Z EBV replication activator: ZEBRA also known Z, Zta, or EB1) acts as a master regulator of the transition from the latent to the lytic replication cycle in latently infected cells. ZEBRA can transactivate both early and late EBV genes, thereby inducing the lytic cycle cascade [2,27,28]. In the latent stage of infection, the ZEBRA protein is not detectable, and only a limited group of viral genes is expressed [28]. It has become clear that post-translational modification of DNA-associated histone by HAT and HDAC in the BZLF1 promoter plays an important role in the maintenance and disruption of EBV latency [27]. EBV latency is primarily maintained by hypoacetylation of histones in the BZLF1 promoter by HDACs. For example, the cellular Sp1/Sp3 protein complex and myocyte enhancer binding factor-2 are associated with HDAC molecules such as HDAC1, 2, 4, 5, and 7, and they recruit these molecules to the BZLF1 promoter [29,30,31]. These complexes lead to hypoacetylation of local histones and establishment of transcriptional latency. However, we have additionally found another mechanism by which EBV latency is maintained in B95-8 cells; the histone H3 lysine 9 (H3K9) methyltransferase catalyzes trimethylation at H3K9 (H3K9 me3), and H3K9 me3 epigenetic repression are involved in BZLF1 transcriptional silencing [32]. Likewise, Murata et al. have reported trimethylation at different histones such as H3K27 me3 and H4K20 me3 for the latency of infected EBV in other cells such as Raji and lymphoblastoid cell lines [33]. These findings clearly indicate that in addition to deacetylation, methylation is an important epigenetic modulation for the maintenance of EBV latency.

Reactivation of latent EBV is associated with progeny virus production and several life-threatening diseases [1]. Therefore, elucidation of the mechanisms that promote or disrupt EBV latency is required to understand the pathobiology of EBV infection and to develop preventive measures or novel therapies against it. EBV becomes latent within B lymphocytes and epithelial cells, which are important cellular constituents of the periodontium. Thus, it is plausible that “on” and “off” switching is repeated daily within these cells in the oral cavity.

BZLF1 transactivation is induced by a variety of stimuli including HDAC inhibitors, 12-O-tetradecanoylphorbol-13-acetate, anti-immunoglobulin, and calcium ionophore [2,27,34]. Among these, butyric acid (BA) is one of the HDAC inhibitors known to exert the highest stimulation [35]. BA belongs a short-chain fatty acid (SCFA) family that consists of acetic acid (AA: C2), propionic acid (PA: C3), BA (C4), and valeric acid (C5). These SCFAs are produced by the bacterial fermentation of dietary fiber [36]. The highest concentration of SCFAs in the body is found in the colon. Most SCFA studies have therefore been conducted with the intestinal microflora of human and animal hindguts [36]. These SCFAs are produced by bacteria during anaerobic glycolysis; pyruvic acid produced through the tricarboxylic acid cycle is broken into various SCFAs depending on the kinds of available catalytic enzymes to synthesize NADPH in the absence of oxygen [36]. Among SCFAs, BA alone inhibits HDAC activity [35]. Given this connection, the fact that some periodontopathic bacteria produce BA is of a particular importance in the periodontium [5,6,34]. Although mechanisms for maintaining EBV latency have been clarified in cell culture, those required to reactivate latent EBV in vivo have not yet been elucidated.

## 3. Microbial Interaction Between Periodontopathic Bacteria and EBV May Control EBV Reactivation in the Periodontium

EBV reactivation is the inevitable step for the onset of EBV-related infectious diseases. However, the precise mechanism that regulates the switch between latent and lytic replication and the trigger molecules responsible for turning on and off this switch in vivo remain poorly understood, and this is a central problem in our understanding of EBV pathogenesis. Some reports have demonstrated HIV-1 replication to be induced by co-infection with a variety of microbes commonly found during HIV-1 infection including *Mycobacterium* species, *Candida albicans*, human T-lymphotropic virus type 1, herpesviruses, and *Peptococcus* species [37]. From a similar viewpoint, previous reports have demonstrated that co-infection with EBV and other pathogens such as malaria and HIV in EBV-infected individuals is associated with increased EBV replication [38,39]. In terms of periodontal pathogenicity, because chronic periodontitis is believed to be caused by endogenous bacteria, the interaction between virus and bacteria co-infection may be of greater importance. To date, however, no report describes such co-infection relating to EBV reactivation in the periodontium.

**Figure 2:**
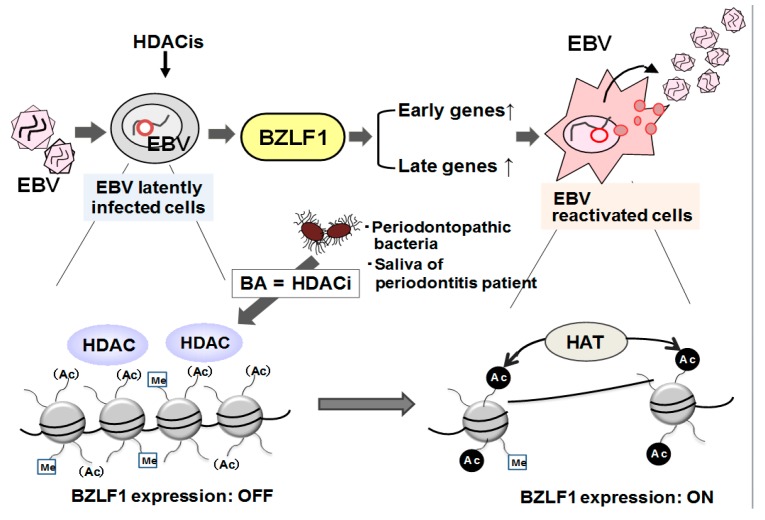
Induction of the EBV lytic switch transactivator BZLF1 involving epigenetic regulation. The latent form of EBV can be induced to enter the lytic replication cycle by treatment with various inducers such as HDAC inhibitors (e.g., BA and trichostatin A), TPA, and antibodies against immunoglobulins. These inducers lead to increased transcription of the immediate–early EBV gene, BZLF1, which encodes ZEBRA. In the latent state, hypoacetylation of histone proteins in the BZLF1 promoter by HDACs is primarily involved in the maintenance of EBV latency. Upon cellular stimulation, local histones are acetylated, the negative regulators are dismissed together with HDAC proteins, and BZLF1 transcription is initiated. BZLF1 can transactivate both early and late EBV genes, thereby inducing the lytic cycle cascade. Replicated EBV infects other cells, thereby further spreading the EBV infection. Periodontopathic bacteria such as *P. gingivalis* and *F. nucleatum* produce high concentrations of BA. Thus, periodontopathic bacteria can induce BZLF1 expression by stimulating acetylation of histones and HDAC dissociation from the BZLF1 promoter in latently infected cells, and BA may be responsible for this effect. BA, butyric acid; BZLF1, BamHI Z fragment leftward open reading frame 1; EBV, Epstein–Barr virus; HDAC, histone deacetylase; ZEBRA, BamHI Z EBV replication activator.

Niederman et al. previously reported that high concentrations of BA have been demonstrated in crevicular fluid exudate from periodontal pockets [40]. Because HDAC contributes to the maintenance of EBV latency and BA, as a quite efficient HDAC inhibitor, is involved in untying the repressed chromatin [27], we have hypothesized that because *P. gingivalis* is a BA-producer, *P. gingivalis* is able to reactivate EBV in the periodontium. We have observed that *P. gingivalis* clearly induces ZEBRA expression at the transcriptional level [34]. Because no such activity is found with the *P. gingivalis* cell alone or several of its individual components such as lipopolysaccharide or fimbriae, this activity is most likely ascribable to BA in the bacterial culture supernatant, which results in lysine acetylation of histone H3 in the BZLF1 promoter. Although *P. gingivalis* produces several SCFAs, we have found that BA alone accelerates ZEBRA induction and histone H3 acetylation in EBV-infected cells (Figure 2) [34].

In addition, much greater amounts of BA are produced by another periodontopathic bacterium, *F. nucleatum* [5,34]. As expected, culture supernatants of *F. nucleatum* induce ZEBRA expression and lysine acetylation of histone H3 in a manner similar to that of *P. gingivalis* [34]. Our findings thus indicate that H3 histone acetylation and ZEBRA induction is ascribable to BA in bacterial culture supernatants. In a chromatin immunoprecipitation assay, we have observed that HDAC1, 2, and 7 are present in the core BZLF1 promoter region (from −176 to + 61) but are dissociated from the promoter concomitantly with acetylated histone H3 upon stimulation with *P. gingivalis* culture supernatant [34]. These observations suggest that *P. gingivalis* and *F. nucleatum* play a role as inducers of EBV reactivation by stimulating histone acetylation and HDAC dissociation from the BZLF1 promoter in latently infected cells.

Previous studies support our hypothesis that the concentrations of BA in supragingival dental plaques of school- and college-aged people (4.7–13.8 mM) [41,42], in gingival crevicular fluids of patients with chronic periodontitis (2.6 ± 0.4 mM) [40], and, surprisingly, in some saliva samples of dental patients during their oral hygiene maintenance (0–2.94 mM) [43] are more than sufficient to induce viral reactivation [34]. By contrast, concentrations of BA are below detection limits in gingival crevicular fluids collected from periodontally healthy sites, even in patients with chronic periodontitis [40]. In addition, several studies have shown activation of ZEBRA expression and lytic EBV replication following intraperitoneal injection of BA in some EBV tumors in vivo [44,45]. These observations imply that BA has a significant role in EBV reactivation in individuals with latent EBV infections, and, therefore, may contribute to the onset and clinical progression of EBV-related diseases. Because sufficient concentrations of BA have been detected in the crevicular fluid in the periodontal pocket as described above, such concentrations of BA should also be detectable in the saliva of patients with chronic periodontitis. In terms of periodontal pathogenicity, BA in the saliva should be capable of reactivating latent EBV. However, no available report yet evaluates BA levels in patients with chronic periodontitis.

Therefore, we have recently conducted a small-sized survey to examine whether BA in the saliva of patients with chronic periodontitis is able to reactivate EBV in latently infected cells in vitro as a pilot study [46]. This has revealed for the first time that there are significantly higher levels of BA in the saliva of patients with chronic periodontitis, and the saliva has efficiently induced BZLF1 transcription. We have furthermore observed that high concentrations of BA, AA, and PA are present in the saliva of patients with chronic periodontitis. However, a significant correlation only occurred between BA concentrations and the levels of BZLF1 transcripts (r = 0.88; *p <* 0.02) [46]. These results support our previous findings that among several SCFAs in the culture supernatant of periodontopathic bacteria, only BA has reactivated EBV [34]. In addition, because BA is one of the most efficient inhibitors of HDACs, and our previous report indicates that BA in the culture supernatant of periodontopathic bacteria promotes histone acetylation and the transcriptional activity of the BZLF1 gene [34], we have further examined whether the saliva samples of patients with chronic periodontitis induce histone acetylation. We have found that the saliva of patients with chronic periodontitis indeed induces lysine acetylation of histone H3 in EBV-positive cells [46]. Although it is necessary to assess other factors contained in saliva such as cytokines and enzymes, our findings suggest that H3 histone acetylation and BZLF1 expression are ascribable to BA in the saliva of patients with chronic periodontitis. This further suggests that BA in the saliva of patients with chronic periodontitis may be involved in the progression of EBV-related diseases including chronic periodontitis. In this context, latent human herpesvirus 8, known as Kaposi sarcoma-associated herpesvirus has also been reactivated by the saliva of patients with chronic periodontitis mainly due to BA [47].

EBV is reactivated through epigenetic modulation, which results in progeny viruses in large quantities in the periodontium affected by chronic periodontitis. However, periodontal pathogenicity of EBV has not yet been well elucidated.

## 4. Etiological Significances of EBV Infection in the Periodontium

Few studies are thus far available that describe de novo etiological significance for the onset or progression of chronic periodontitis with latent EBV infection in the periodontium. We therefore introduce herein some progress in understanding EBV-related periodontal etiology from Doglio’s group and ours [17,48,49].

Although B lymphocytes have been recognized as host cells for EBV infection in humans, Vincent-Bugnas et al. have recently revealed that epithelial cells of the periodontium (pECs) are commonly infected with EBV. They have proposed that the pECs may serve as an important oral reservoir of cells latently infected with EBV [17]. Using a simple, non-surgical tissue-sampling procedure, they have found frequent EBV infection in the periodontal epithelium that surrounds and attaches teeth to the gingiva. In addition to detection of EBV-specific latent transcripts such as EBV-encoded latent membrane proteins 1 and 2 (LMP1 and LMP2) and EBV-encoded nuclear antigen 1 and 2 (EBNA1 and EBNA2), as well as lytic transcripts such as BZLF1, they have additionally revealed that the basal level of epithelial EBV-infection is significantly increased in chronic periodontitis [17]. Moreover, in accordance with the levels of clinical attachment loss, the frequency of EBV-encoded small nuclear RNA (EBER)-positive pECs in the gingival sulcus, and EBNA1 expression in the sulcus, the level of EBV infection correlates with periodontal disease severity [17]. Fluorescent terminal deoxynucleotidyl transferase-mediated dUTP nick-end labeling (TUNEL) assay in conjunction with immunofluorescent staining for LMP2 revealed that EBV-infected pECs in inflamed tissues appear to be prone to apoptosis. In addition, using a histological analysis combining EBER-in-situ hybridization and chemokine staining showed that those cells produce greater amounts of CCL20, a pivotal inflammatory chemokine controlling immune cell infiltration into tissues [17]. Moreover, CCL20 RNA is highly produced in diseased subjects but not in healthy donors, and the level of CCL20 RNA expression correlates closely with that of EBNA1. Their discovery that the periodontal epithelium is a major site of latent EBV infection sheds a new light on EBV persistence in healthy carriers and on the role of this ubiquitous virus in chronic periodontitis. Moreover, the identification of this easily accessible site of latent EBV infection may encourage new approaches to investigate and monitor other EBV-associated disorders [48].

To further investigate mechanisms pertaining to pathogenesis of EBV latently infected periodontium, we recently examined potential periodontal pathogenesis associated with LMP1 [49]. C-terminal activating region (CATR) domains of LMP1 bind to TNF receptor-associated factor and TNF receptor-associated death domain protein. This binding is closely related to the activation of nuclear factor kappa B (NF-κB) and consequently induces the production of pro-inflammatory cytokines such as interleukin-8 (IL-8, CXCL8). As expected, we have found that LMP1 induces IL-8 production in Ca9-22 human gingival cells in terms of IL-8 mRNA expression, NF-κB transcription, IL-8 protein production, and the phosphorylation of NF-κB p65 with inhibitor of kappa B alpha (IκBα), respectively [49]. In addition, to identify sequences of LMP1 responsible for the activation of NF-κB, we have used two LMP1 mutants lacking the CATR domains that activate NF-κB. We found that extremely high IL-8 protein production is induced by LMP1 in a time- and dose-dependent manner, where simultaneous phosphorylation of NF-κB p65 and IκBα and transcription of NF-κB are observed [49]. By contrast, IL-8 production and NF-κB transcription are drastically inhibited by a dominant-negative mutant of IκBα. Moreover, the LMP1 mutants fail to induce IL-8 protein production. Our results suggest that due to its CATR domains, LMP1 contributes to the onset or progression of chronic periodontitis via IL-8 production attributable to NF-κB activation, which may contribute to clarify a mechanism of pathogenesis associated with latent EBV in chronic periodontitis. However, from a reverse viewpoint, very few mechanisms as described above are so far delineated. Therefore, how EBV, irrespective of its activation status, relates to the onset or progression of chronic periodontitis as a causative agent remains to be investigated.

In this regard, EBV was recently suggested to be involved in the pathogenesis of other chronic inflammatory infections occurring in the periodontium such as peri-implantitis and periapical periodontitis [50,51,52]. In contrast with unexplained peri-implantitis, some interesting mechanisms are reported with periapical periodontitis, i.e., BA excreted by *P. endodontalis* and *F. nucleatum* in addition to EBV is involved in the progression of periapical periodontitis [53,54]. An underlying mechanism may involve reactive oxygen species released from EBV-infected host cells that induce local imbalance between receptor activator NF-κB and osteoprotegerin, which consequently promotes alveolar bone resorption [55].

## 5. Concluding Remarks

Our aforementioned research may have provided evidence for a possible microbial interaction between EBV and some periodontopathic bacteria in chronic periodontitis etiology. Because the regulation of the switch from latency to reactivation is an initial key step in EBV infection, our observations suggest that BA-producing periodontopathic bacteria have the potential to trigger EBV reactivation in the periodontium of EBV-infected individuals. In addition, inflammatory cytokines such as IL-1, IL-6, and IL-8 play an important role in chronic periodontitis etiology, and increased concentrations of cytokines in sera from EBV-infected patients have been reported [12,56]. The envelope protein and genomic DNA of EBV can stimulate inflammatory cytokines in primary human monocytes [57,58]. We also have found that EBV protein, which is produced only during the lytic phase, induces greater activation of NF-κB and production of IL-6 and IL-8 from human gingival fibroblasts when compared with the stimulation of LPS by *P. gingivalis* (unpublished data). These observations suggest that EBV is intimately interrelated with the onset and various progression stages of chronic periodontitis.

**Figure 3:**
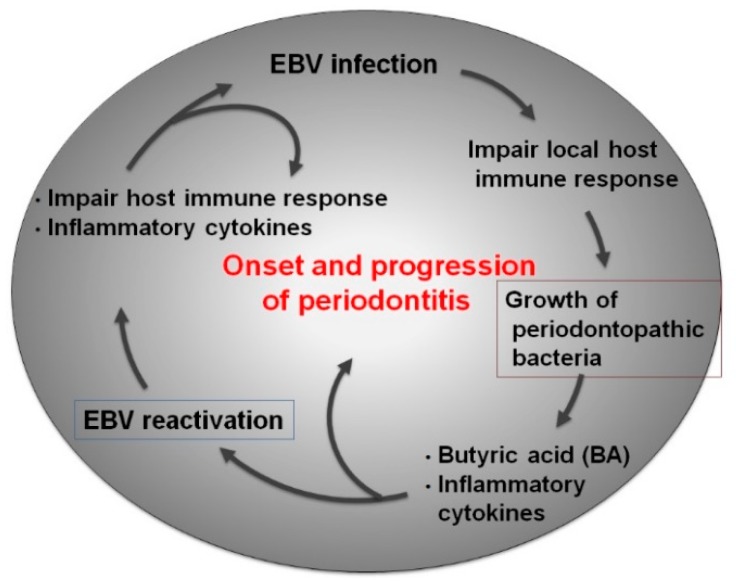
Interactions between bacteria and viruses in the oral cavity. The oral cavity is colonized by a wide variety of and numerous microbes, including oral bacteria, viruses, and fungi. Thus, in addition to host–microbial interactions, the interactions of herpesviruses such as EBV with periodontopathic bacteria have the potential to contribute to periodontitis pathogenesis.

We therefore assume that microbial synergy by the interaction between BA-producing periodontopathic bacteria and EBV leads to the following negative chain of etiological events in the periodontium (Figure 3): (1) periodontopathic anaerobic bacteria such as *P. gingivalis* and *F. nucleatum* produce BA; (2) BA induces EBV reactivation; (3) reactivated EBV induces production of inflammatory cytokines from the host cells, which causes local inflammation and consequently disturbs local host defenses; (4) this accelerates periodontopathic bacterial infection; (5) this exacerbates inflammatory cytokine production by the synergistic effects of EBV and periodontopathic bacteria; and (6) this collectively advances chronic periodontitis.

Chronic periodontitis and EBV are spreading worldwide. This review delineates a causal relation between EBV reactivation and BA in oral fluids including the gingival crevicular fluid and saliva of patients with chronic periodontitis (probably ascribable to infection of BA-producing periodontopathic bacteria such as *P. gingivalis* and *F. nucleatum*). Despite this, further basic studies with additional points of view on etiological mechanisms and clinical studies with greater numbers of cases are needed. However, it should be noted that EBV displays species-specificity with the human as its only known host; because it infects neither mice nor rats, advancements in this field have been limited. In this connection, murine herpesvirus 68, a gamma herpesvirus closely related to EBV, was reported for the first time recently [59]. We therefore have been developing a new experimental system using humanized mice. Thus far, these mice show infiltration of EBV-positive B lymphocytes and the induction of osteoclasts in the periodontium (unpublished data). Validation of this system is currently in progress.

**Figure 4:**
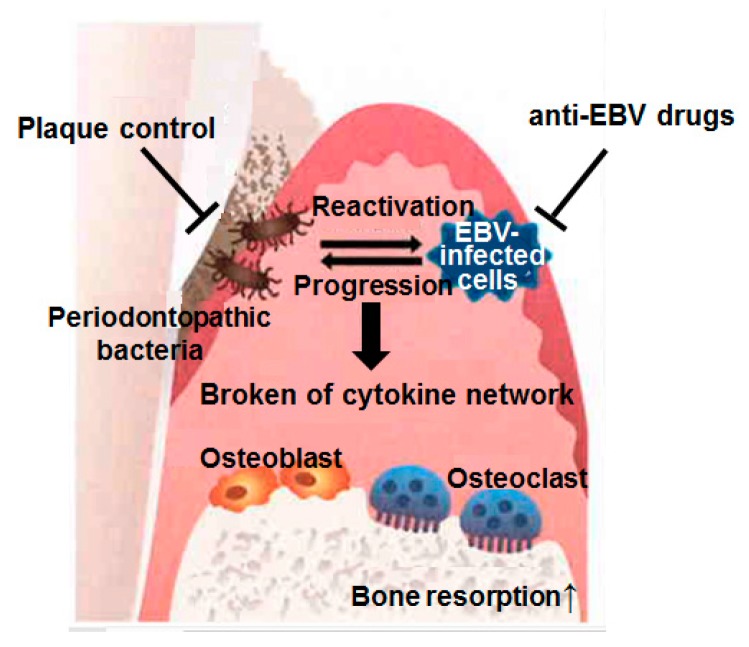
Microbial synergy by EBV–bacterial interaction in periodontitis pathogenesis. There is a possibility that a “negative chain reaction” by EBV and periodontopathic bacteria contributes to the etiology of severe periodontitis. It is expected that future basic clinical studies will determine whether the concept of a “periodontopathic virus” is applicable to the etiology of periodontitis.

At this moment, even with a primitive understanding of EBV pathogenicity for chronic periodontitis, prevention and early treatment of chronic periodontitis targeting the elimination of BA-producing bacteria dwelling in the periodontium seems effective for blocking further clinical progression of EBV infection including chronic periodontitis itself (Figure 4).

Thus, an ideal approach for periodontal treatment when taking EBV infection into account as a cause may involve the following: (1) perform conventional plaque control for preventing reactivation of latent EBV as well as reducing BA-producing periodontopathic bacteria; and (2) prescribe anti-herpes drugs to interrupt the production of progeny EBV in the affected periodontium.
